# Multi-Omic Regulation of the PAM50 Gene Signature in Breast Cancer Molecular Subtypes

**DOI:** 10.3389/fonc.2020.00845

**Published:** 2020-05-22

**Authors:** Soledad Ochoa, Guillermo de Anda-Jáuregui, Enrique Hernández-Lemus

**Affiliations:** ^1^Computational Genomics Division, National Institute of Genomic Medicine, Mexico City, Mexico; ^2^Graduate Program in Biomedical Sciences, Universidad Nacional Autónoma de México, Mexico City, Mexico; ^3^Cátedras Conacyt para Jóvenes Investigadores', National Council on Science and Technology, Mexico City, Mexico; ^4^Center for Complexity Sciences, Universidad Nacional Autónoma de México, Mexico City, Mexico

**Keywords:** multi-omic approaches, breast cancer subtypes, PAM50, elastic net, data integration

## Abstract

Breast cancer is a disease that exhibits heterogeneity that goes from the genomic to the clinical levels. This heterogeneity is thought to be captured (at least partially) by the so-called breast cancer molecular subtypes. These molecular subtypes were initially defined based on the unsupervised clustering of gene expression and its correlate with histological, morphological, phenotypic and clinical features already known. Later, a 50-gene signature, PAM50, was defined in order to identify the biological subtype of a given sample within the clinical setting. The PAM50 signature was obtained by the use of unsupervised statistical methods, and therefore no limitation was set on the biological relevance (or lack of) of the selected genes beyond its predictive capacity. An open question that remains is what are the regulatory elements that drive the various expression behaviors of this set of genes in the different molecular subtypes. This question becomes more relevant as the measurement of more biological layers of regulation becomes accessible. In this work, we analyzed the gene expression regulation of the 50 genes in the PAM50 signature, in terms of (a) gene co-expression, (b) transcription factors, (c) micro-RNAs, and (d) methylation. Using data from the Cancer Genome Atlas (TCGA) for the Luminal A and B, Basal, and HER2-enriched molecular subtypes as well as normal tumor adjacent tissue, we identified predictors for gene expression through the use of an elastic net model. We compare and contrast the sets of identified regulators for the gene signature in each molecular subtype, and systematically compare them to current literature. We also identified a unique set of predictors for the expression of genes in the PAM50 signature associated with each of the molecular subtypes. Most selected predictors are exclusive for a PAM50 gene and predictors are not shared across subtypes. There are only 13 coding transcripts and 2 miRNAs selected for the four subtypes. *MiR-21* and *miR-10b* connect almost all the PAM50 genes in all the subtypes and normal tissue, but do it in an exclusive manner, suggesting a cancer switch from *miR-10b* coordination in normal tissue to *miR-21*. The PAM50 gene sets of selected predictors that enrich for a function across subtypes, support that different regulatory molecular mechanisms are taking place. With this study we aim to a wider understanding of the regulatory mechanisms that differentiate the expression of the PAM50 signature, which in turn could perhaps help understand the molecular basis of the differences between the molecular subtypes.

## 1. Introduction

Breast cancer is the most common cause of cancer death among females (1). Breast tumors have been classified in molecular subtypes with distinctive clinical characteristics and a recognizable gene expression signature (2). Such signature has been reduced to 50 genes that achieve the best separation of subtypes, attaining the PAM50 classifier (3). However, the physiological implications of the difference in gene expression, if any, are not well-understood.

Given that gene expression is regulated by several interconnected mechanisms (4–7), differences across subtypes are expected for these mechanisms. Evidence of this was found in the form of distinguishable patterns of DNA methylation, mutation and miRNA expression that shape groups partially equivalent to the molecular subtypes (8). These patterns imply a link between the different omics and PAM50 gene expression, but do not clarify which genomic, epigenetic or post transcriptional changes drive the expression signature of such molecular subtypes. To advance in the identification of such drivers of molecular subtypes expression, we propose the use of a sparse model of PAM50 gene expression.

Sparse models achieve the selection of the best predictors of an independent variable by fitting penalized linear models. The penalization of the regression coefficients aim is to shrink them toward zero in such a way that predictors contributing lowly to prediction i.e., poorly associated with the independent variable, end up with null coefficient values and get filtered out of the model (9). Ridge Regression, Least Absolute Shrinkage and Selection Operator, and Elastic Network methods apply different penalizations. The elastic network approach selects groups of pairwise correlated variables instead of choosing a single predictor from the group (10, 11), augmenting the space of predictors of interest but also incrementing false positive rates (12).

Sparse models have been proposed for multi-omic sample classification (13, 14) and biomarker identification (15–17); but their capacity to simplify multi-omics co-interpretation has only been tested in the evaluation of the extent of different omics effects over a phenotype (18, 19). Here, the predictor selection capability of the elastic network approach is exploited to identify the CpGs, coding transcripts, and miRNAs most associated with the expression of the PAM50 genes in order to outline molecular differences behind the gene expression patterns characterizing breast cancer subtypes within a true multi-omic framework. The hypothesis is that PAM50 gene expression patterns are accompanied by distinctive regulatory elements, reflecting the way gene expression is controlled in the different breast cancer subtypes.

## 2. Methods

### 2.1. Data Acquisition

Concurrent experimental samples of DNA methylation, transcript and miRNA expression were downloaded from the GDC (https://portal.gdc.cancer.gov/repository) at May 2019. Only samples with Illumina Human Methylation 450, RNA-seq and miRNA-seq measures were kept; filtering out samples quantified with the Illumina Human Methylation 27 BeadChip, which covers a smaller portion of the genome than the one we wanted to target. Subtype classification was also downloaded from the GDC trough TCGABiolinks R package (20).

After preprocessing them according to Aryee er al. (21), Tarazona et al. (22), and Tam et al. (23), and biomaRt v95, values of methylation for 384,575 probes and expression for 16,475 coding transcripts and 433 miRNA precursors were obtained for 45 unique samples of Her2, 395 LumA, 128 LumB, and 125 Basal subtypes, plus 75 samples of non-tumor (normal adjacent) tissue.

### 2.2. Elastic Network Implementation

The three different data types were concatenated and normalized to have mean = 0 and standard deviation = 1. Eighty percent of the samples for each subtype were used for training, leaving the rest for testing as in Liu et al. (13). Using the R package glmnet (24), elastic network models were fitted per subtype for each gene in the PAM50 classifier with the linked script https://github.com/CSB-IG/PAM50multiomics/blob/master/enetGLMNET.R. The mixing parameter was held fixed at 0.5 because such value has shown a good performance (10), but shrinkage parameter (λ) was optimized between values from 0.001 and 1,000 through repeated cross-validation.

Cross-validation was repeated 100 times with k = 3-folds for the subtypes with <100 training samples (Her2+ subtype and normal tissue) and k = 5 for the more represented subtypes (Luminal A, Luminal B, and Basal). Chosen λ parameters were used to predict testing data and root mean squared error (RMSE) was calculated per model. Fitting was repeated with the same specifications, for only 40 samples per subtype to verify the effect of data set size.

### 2.3. Omics Comparison

For each PAM50 gene model, RMSE was calculated for the testing data either with (1) the complete set of selected predictors, (2) only with selected CpGs, (3) just with selected coding transcripts, or (4) solely with selected miRNAs. Omic's specific RMSE were evaluated by zeroing all coefficients not associated to the omic of interest in the already fitted models with the linked script https://github.com/CSB-IG/PAM50multiomics/blob/master/RMSEperOmics.R, in an approach similar to the one used by Setty et al. (25) to search for key regulators. Obtained values shape RMSE distributions per omic which were compared via Kolmorogov–Smirnov test. This was done both per subtype per omic and mixing all the subtypes in a distribution per omic. *P*-values obtained were corrected for multiple testing with the FDR method.

### 2.4. Test vs. Reported Links Between Predictors and PAM50 Genes

Enrichment for previously reported regulatory links between PAM50 genes and CpGs, TFs, and miRNAs were tested by simple Fisher's Exact Test. Tests repeated by subtypes had *p*-values adjusted by FDR. Regulatory targets were taken from Illumina's annotation in the case of CpGs and from databases accessible through R packages in the case of TFs and miRNAs, with the linked script https://github.com/CSB-IG/PAM50multiomics/blob/master/validateInteractions.R. tftargets https://github.com/slowkow/tftargets is the package used to retrieve TF targets. It queries both predicted and validated data from TRED(2007), ITFP(2008), ENCODE(2012), and TRRUST(2015) databases at the date specified in parentheses next to each resource, plus the lists curated by Neph et al. (26) and Marbach et al. (27).

The package used to retrieve miRNA targets is multiMiR v2.2 (28), it queries DIANA-microT-CDS, ElMMo, MicroCosm, miRanda, miRDB, PicTar, PITA, TargetScan, miRecords, miRTarBase, and TarBase, also reporting both experimentally validated and predicted results. Universe size for enrichment tests were taken from these databases, constrained to regulators measured in the input datasets. The hypothesis is that models selected reported associations between a PAM50 gene and a regulator measured in the input dataset more than expected.

### 2.5. Analysis of the Selected Predictors

Selected predictors and associated coefficient values were loaded to Cytoscape to construct a network of PAM50 gene predictors per subtype. PAM50 genes are taken as targets while predictors are sources, this makes a directed network were out and indegree are estimated. Predictors with the largest outdegree were submitted to an analysis of differential expression and their coefficient value distributions were compared to the global miRNA distribution via Kolmorogov–Smirnov tests. The differential analysis of miRNA expression was done per subtype by limma's package *treat* function in order to control for both fold change and significance (29). A minimum fold change of 1.1 was used.

### 2.6. Gene Enrichment Analysis

Every set of predictors selected for a PAM50 gene was submitted to functional enrichment analysis with the R package *HTSanalyzeR* v2.13.1 (30) versus the GO-BP with the linked script https://github.com/CSB-IG/PAM50multiomics/blob/master/enrichment.R. Sets enriched across subtypes were further tested via Fisher's Exact Test with the alternative hypothesis that selection in one subtype is exclusive with regards to selection another subtype.

**Figure 1:**
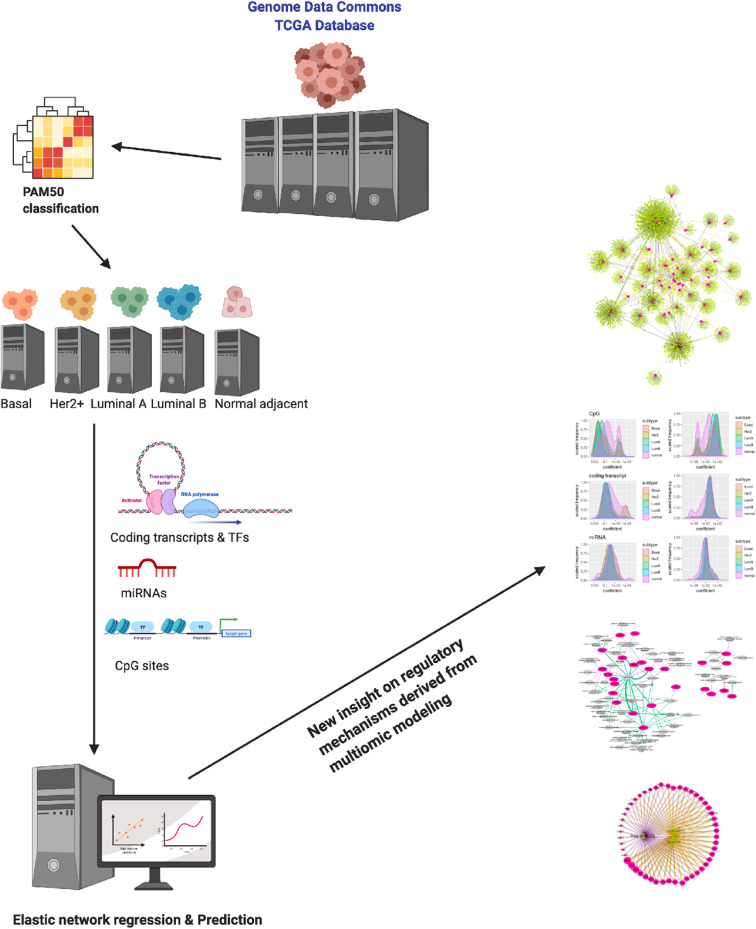
Schematic depiction of this work. By analyzing multiomic data from the TCGA/Genome Data Commons collaboration for the different breast cancer molecular subtypes and healthy adjacent breast tissue via a generalized elastic network modeling, we have been able to derive some insight on the way the PAM50 genes are regulated (as predicted by the model). Results may shine some new light on the way PAM50 genes are able to capture intrinsic features of these phenotypes.

The code to perform all previous analyses (see Figure 1) can be found at the following GitHub repository: https://github.com/CSB-IG/PAM50multiomics

## 3. Results

Elastic network models were fitted per gene, regressing PAM50 gene expression to DNA methylation, miRNA and coding transcript expression. Elastic networks model shrink the regression coefficients toward 0, filtering predictors by its strength of association with the variable of interest. This ability for feature selection was exploited versus unfiltered omic data to identify the CpGs, coding transcripts and miRNAs most related to the PAM50 genes in cancer subtypes and normal tissue.

We fitted five models for each PAM50 gene, one per subtype and one for the normal tissue, since differences are expected for each of the 5 phenotypes. Descriptors of models per subtype and omic are reported in Table 1.

**Table 1:** Size of input and output of the models per subtype: Basal, Her2+, Luminal A, Luminal B as well as normal (i.e. tumor-adjacent healthy tissue).

	**Basal**	**Her2+**	**LumA**	**LumB**	**Normal**
Samples	125	45	395	128	75
Selected CpGs	3,090	2,514	7,173	1,485	5,373
Known CpGs selected	9	0	21	12	0
Selected coding transcripts	1,525	591	3,115	888	2,340
Selected TFs	207	91	465	133	327
Selected TFs predicted by another software	15	2	49	11	19
Selected TFs experimentally observed	4	3	25	7	9
miRNAs	101	85	174	116	123
Selected miRNAs predicted by another software	7	3	7	2	4
Selected miRNAs experimentally observed	8	5	8	12	5

**Figure 2:**
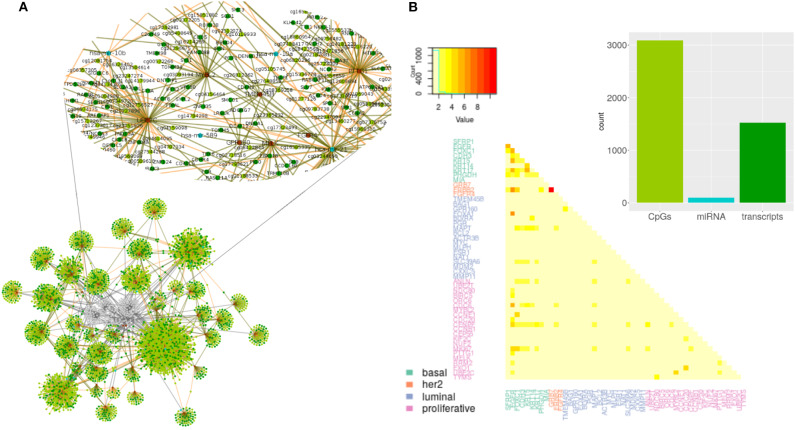
Predictors selected per PAM50 gene for Basal subtype. **(A)** Topology of selected predictors and associated PAM50 genes. Brown circles are PAM50 genes. Transcripts are colored in dark green, miRNAs in blue, and CpGs in light green. Edges link each PAM50 gene with its selected predictors. The color of the line indicates the sign of the coefficient value associated with the predictor; negative values are in brown and positive ones in green. Zoom of the gray area shows the predictors selected for *MYBL2*. **(B)** Summary of the network. Barplot with the total representation of each omic plus heatmap of the count of predictors shared by PAM50 genes.

The output of the model are lists of associations between PAM50 genes and the selected predictors. Each selected predictor has a coefficient of regression whose value reflects the extent of association with the PAM50 gene. Coefficients are never zero, since this value means predictors can be filtered out of the prediction; but can be both negative and positive indicating an opposite effect over the predicted value. Lists of associations shape networks like the one represented in Figure 2. Networks for the other subtypes and the normal tissue can be found at Figures S1–S4.

From observation of networks of selected predictors to PAM50 genes, it is evident that CpGs are the most selected predictors, followed by transcripts and with only a few miRNAs selected. It can also be seen that most predictors are exclusive of a PAM50 gene but all the PAM50 genes share predictors whose pattern of expression or methylation links one gene to another. This suggests the complete set of PAM50 expression is coordinated, independently of the gene being of luminal expression, basal, or any other signature.

### 3.1. Omics Contribute Differently to PAM50 Gene Expression Prediction in Normal Tissue and Cancer

In order to test the reliability of the fitted models, we checked the prediction error and the selection of previously reported associations. Regulation through DNA methylation, miRNA, or TF targeting is hence regarded as true positive and compared to model's results.

**Figure 3:**
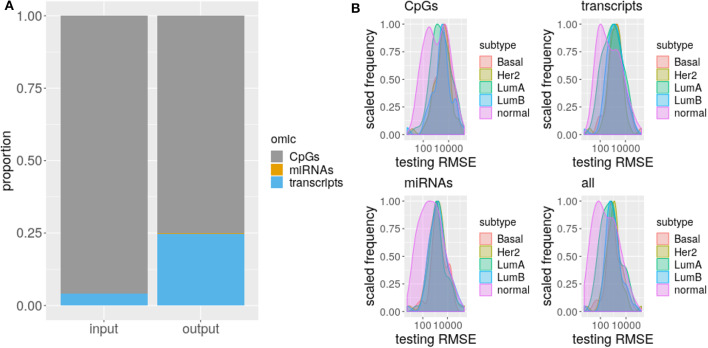
Omics differ on selection and RMSE. **(A)** The proportion of each omic is shown for input and selected sets. The different omics are represented by a different color. **(B)** Distribution of testing RMSE per subtypes are displayed for single and multi-omics.

The proportion of selected predictors can not be explained solely by the size of the omics taken as input (χ^2^, *p*-value <2.2e-16, Figure 3), specifically, coding transcripts and miRNAs are overrepresented in the models (Fisher's Exact Test, *p*-value <2.2e-16). Concordantly, there are more true TF (Fisher's Exact Test, *p*-value ≤ 1.942846e-05) and miRNA (Fisher's Exact Test, *p*-value ≤ 7.573200e-11) relations than expected but less CpGs (Fisher's Exact Test, *p*-value ≤ 4.311267e-03). The exception is LumB subtype which has as many true positive CpGs as expected.

Given the difference between input and selected proportion of omics, we hypothesized a discrepant prediction power of CpGs, coding transcripts, and miRNAs. To test this, we evaluated models carrying the complete set of selected predictors or just the predictors from each omic.

As RMSE is a standard measure to compare regression models that measures how far is the model prediction from the observed data in response variable units, then, the lower its value the better. Normally, the error decreases the more independent predictors are included in the model, so we choose not to fit again with the selected predictor per omic, but to test the exact same model with the jointly fitted coefficient values, just zeroing predictor's coefficients from other than the omic of interest. This way, the RMSE distribution of a model containing only predictors of a given omic, represents how much of the total prediction is contributed by the predictors from that omic.

As suggested by the difference with the input proportions, DNA methylation is the less predictive omic for all the subtypes, thought this difference is not always significant (CpGs vs. coding transcripts ks. test *p*-value ≤ 0.03192 for LumB, Her2+, and Basal and CpGs vs. miRNAs ks. test *p*-value ≤ 0.02222 for Her2+ and Basal). This disagrees with the great prediction improvement reported by Huang et al. (16) for methylation data, a fact that could be driven by the much larger and heterogenous input data used here, that we believe captures better the heterogeneity of breast cancer subtypes. Meanwhile, coding transcript and miRNAs contribute the same, with no significant difference between their distributions for all the subtypes.

Remarkably, the error distribution obtained with the complete set of predictors significantly outperforms CpGs and some subtype miRNAs (ks.test *p*-value ≤ 0.02222 for LumA and Basal) but never outweighs coding transcripts. Single omics can not beat multi-omics error due to the design of the test, thus the outperforming of CpGs and miRNAs is unsurprising, what is startling is the complete statistical agreement between multi-omics prediction power and coding transcripts prediction power, which supports gene expression as the current best biomarker of molecular subtypes. We must note however that this may be related to (1) more info on RNA and (2) PAM50 was derived from expression signatures.

Finally, there is no significant difference across subtypes RMSE distributions for both single-omics and multi-omics, but CpGs (ks.test *p*-value ≤ 0.01601952), miRNAs (ks.test *p*-value ≤ 0.002834981), and multi-omics (ks.test *p*-value ≤ 0.03919459) distributions of normal tissue differ from the distribution of each subtype, suggesting these omics represent a distinct amount of PAM50 gene expression in normal tissue than in cancer, that is, the association of DNA methylation and miRNA expression with PAM50 gene expression is altered in cancer.

### 3.2. The Association Strength Distributions of Predictors Are Different for Each Subtype

**Figure 4:**
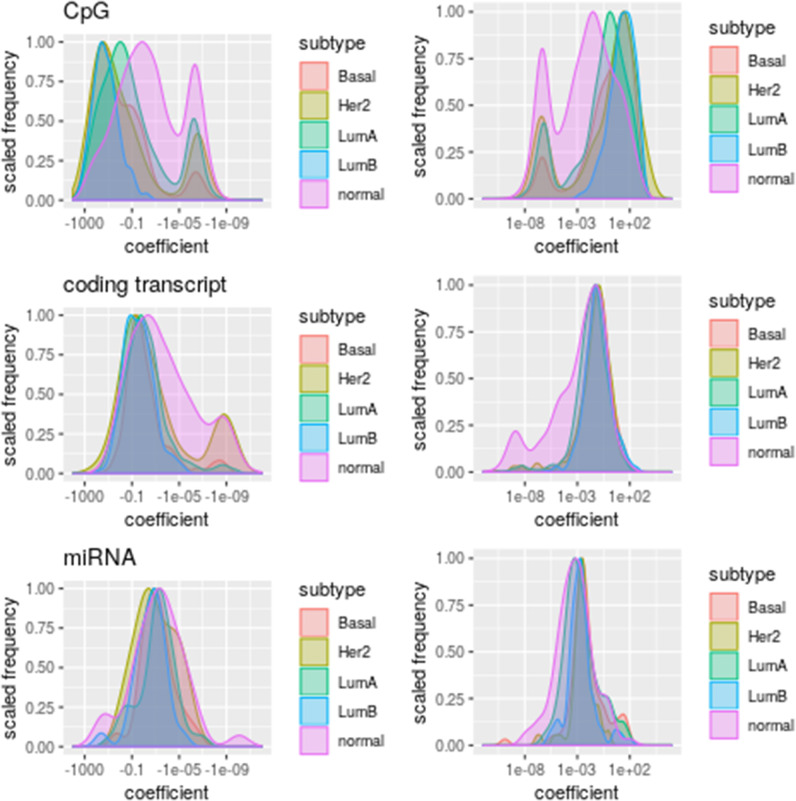
Distribution of coefficient values per omic. The subtypes are represented by different colors. Plots at the **(Left)** column represent negative coefficients whereas those at the **(Right)** column stand for positive coefficient values.

The difference between omics extends to coefficient values, shown in Figure 4. Since coefficients represent the strength of association between predictors and PAM50 expression (16), coefficient values suggest that each omic has a specific association with PAM50 gene expression. Coefficient value distributions are significantly different between subtypes (ks.test *p*-value ≤ 2.82E-02) and omics (ks.test *p*-value ≤ 0.01535) with few exceptions for coding transcripts and miRNAs. Basal, Her2+, and LumB coding transcripts coefficients are not significantly different. Neither are miRNA coefficients of pairs LumA and normal tissue, LumB and Basal subtype, and Basal and Her2.

According to these distributions, DNA methylation has a strong but noisy association with PAM50 gene expression while miRNA (Fisher test *p*-values ≤ 0.001403597) and coding transcript (Fisher test *p*-values ≤ 1.086031e-29) association tends to be positive (Figure S3) and more stable. The elevated association between DNA methylation and PAM50 genes expression explains why so many CpGs get selected in spite of its low prediction power. A stronger association between DNA methylation and gene expression than between gene and miRNA expression had previously been found for ovarian cancer by Sohn et al. (18) using a different penalization modeling.

### 3.3. *miR-21* and *miR-10b* Are the Only Relevant Predictors Selected Across Subtypes

**Figure 5:**
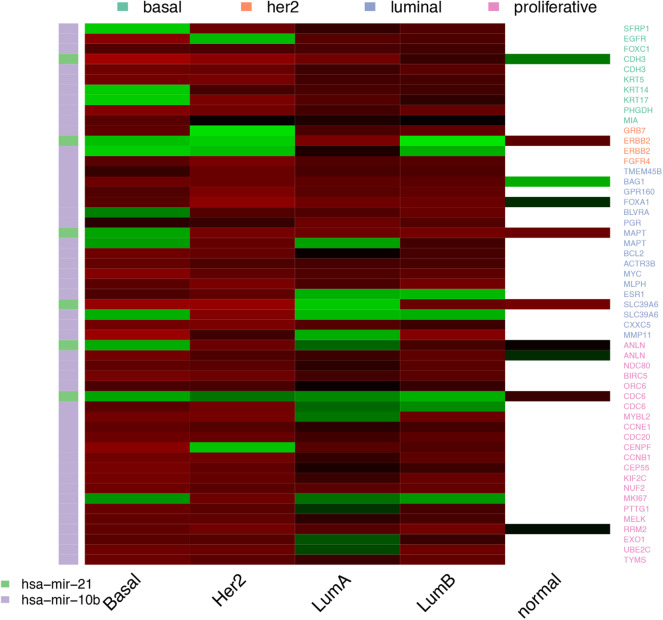
miRNAs selected across the four cancer subtypes. miRNAs are represented by the color bar at the left. The highest positive coefficient value appears in bright red, the lowest negative coefficient is in bright green. Near zero coefficients look black. The white cells in the column of normal tissue means the predictors was not selected.

Next, we wanted to see how variable is actually the association between one predictor and the predicted PAM50 gene, that is, the specific coefficient values, not their distributions. For this, we wanted to focus on the predictors selected for a PAM50 gene across subtypes, shown in Figure 5. However, as noted before, models selected a great quantity of predictors exclusive for each gene, 93.45% of the selected CpGs, 74.24% of the coding transcript, and 81.37% the miRNAs are not shared between any two genes. In consequence, there are no CpGs associated with any gene for all the subtypes but there are 14 relations with coding transcripts and 51 with miRNAs satisfying this.

The 13 coding transcripts selected across subtypes as predictors of a specific PAM50 gene are trivial, since they just portray physical linkage. *ELP2* and *SLC39A6* are coded in opposite strands of the same locus while the rest of pairs are contiguous. Most of the associations, 84.77%, connect a PAM50 gene with a coding transcript in another chromosome, but these are not repeatedly selected across subtypes. It is worth mentioning that although all coefficients values are positive, even close predictors, like *YEATS4* and *SLC35E3* carry distinct coefficients.

Regarding miRNAs, there are only two miRNAs repeatedly selected among subtypes, *miR-10b* and *miR-21*. These are known breast cancer markers targeting some PAM50 genes (31). *Mir-21* has been experimentally linked with *BCL2, MYC, EGFR*, and *ERBB2* expression (32–35) and predicted to target *ESR1* and *FOXA1* (36, 37). On the other hand, *miR-10b* has been linked to *CDC6, EGFR*, and *SFRP1* (38, 39). There is no particular pattern among validated associations or coefficients, other than *miR-21* carrying mostly positive coefficient values and *miR-10b* selection extending up to normal tissue (for the full set of validated interactions please see Supplementary Table S1).

### 3.4. Micro-RNA *miR-21* and *miR-10b* Are Universal PAM50 Predictors in Cancer and Health

Next we wanted to check the role of *miR-21* and *miR-10b* per subtype. With this in mind, we revisited the models derived networks, that link PAM50 genes and predictors per subtype.

The networks show that genes overexpressed in each subtype get larger models. About 30% of the luminal genes have models larger than average for LumA subtype, while almost 90% of basal genes have the equivalent for Basal subtype. Her2+ subtype and normal tissue have no clear pattern, but for LumB subtype, half the luminal genes and 28% of the proliferative ones have increased size models.

Predictors that bridge between PAM50 genes can proceed from any omic, but CpGs are significantly underrepresented (Fisher test *p*-values ≤ 1.81E-88). CpGs are at most, selected for two subtypes as predictors of a specific PAM50 gene. There are just 24 CpGs in this situation, of which 15 are shared between Her2+ and another subtype or the normal tissue, including nine CpGs associated with *ERBB2* but placed in other loci than chromosome 17.

**Figure 6:**
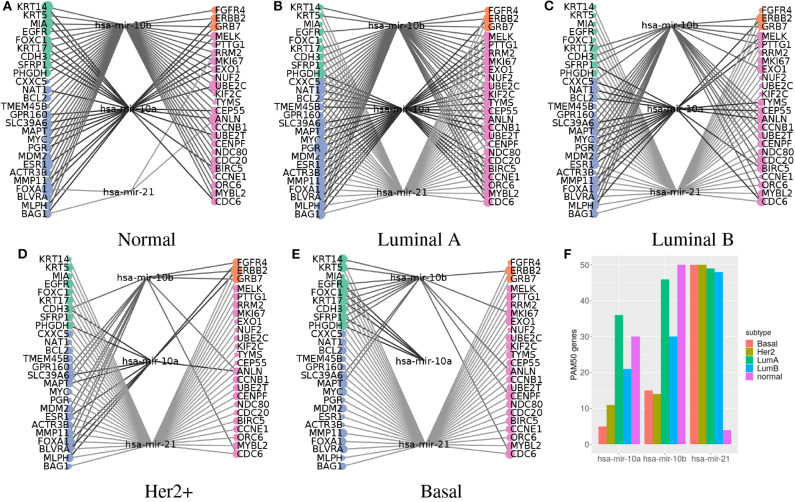
Predictors connecting most PAM50 genes transition from *miR-10a/b* in normal tissue to *miR-21* in cancer subtypes. **(A–E)** PAM50 genes are at the sides, colored according to their pattern of expression: green for basal expression, orange for Her2 enriched, blue for luminal expression, and pink for proliferation favorable. Node size represents the number of predictors selected. Predictors are in the middle. Lines connect PAM50 genes to the associated predictors. Lines are in a gray gradient to distinguish different predictors. **(F)** The bar chart represents the number of PAM50 genes connected to the miRNAs per subtype.

Meanwhile, coding transcripts and miRNAs fulfill this role more often (Fisher test *p*-values ≤ 5.84E-03) than solely input proportions would explain. This is no surprise since both pertain to the same level of molecular features, that of transcripts, as the PAM50 gene expression signature; as such, coding transcript and miRNA may be subject to the same biomolecular pressures. The stunning observation is that one miRNA can link almost all of the PAM50 genes for all the cases (Figure 6). The outstanding miRNAs are again *miR-21* and *miR-10b*.

For normal tissue *miR-10b* was selected as predictor of all PAM50 genes while *miR-21* is linked to only four genes. On the contrary, *miR-21* is connected to most genes in the all the breast cancer subtypes, while *miR-10b* is poorly linked. For LumA subtype, shown in Figure 6B, both *miR-10b* and *miR-10a* are highly connected, but still can not reach genes like *FOXC1*, which is connected instead with *miR-21*.

Both *miR-10a* and *miR-10b* are members of the miR-10 family encoded within the Hox genes genomic clusters; *miR-10a* resides upstream from *HOXB4* and *miR-10b* upstream from *HOXD4* (40). Due to their relatedness they will be referred as *miR-10a/b*.

The hub-like behavior of these miRNAs agrees with previous observations of our group of highly connected miRNAs per subtype (41), which are important for network cohesion (42). Although the coefficients networks maintain a large connected component when removing *miR-10a/b* and *miR-21*, tens to hundreds of predictors are needed to link all the PAM50 genes; when only one of these miRNAs is required to achieve the same.

Given that each miRNA has the potential to target hundreds of genes (43), *miR-10a/b* and *miR-21* are not so exceptional in this regard. However, as explained earlier, only a fraction of PAM50 genes have a regulatory relation with these miRNAs, suggesting most of the detected associations are indirect. Indirectness is consistent with the low values of the coefficients, which range from −0.2938690 to 0.4333184, when miRNAs coefficient values range within two orders of magnitude higher. Coefficient value distributions of *miR-10a/b* and *miR-21* are also significantly different than the rest of miRNA coefficients (ks.test *p*-value ≤ 9.068e-05).

### 3.5. PAM50 Genes Enrich for Different Functions per Subtype

**Figure 7:**
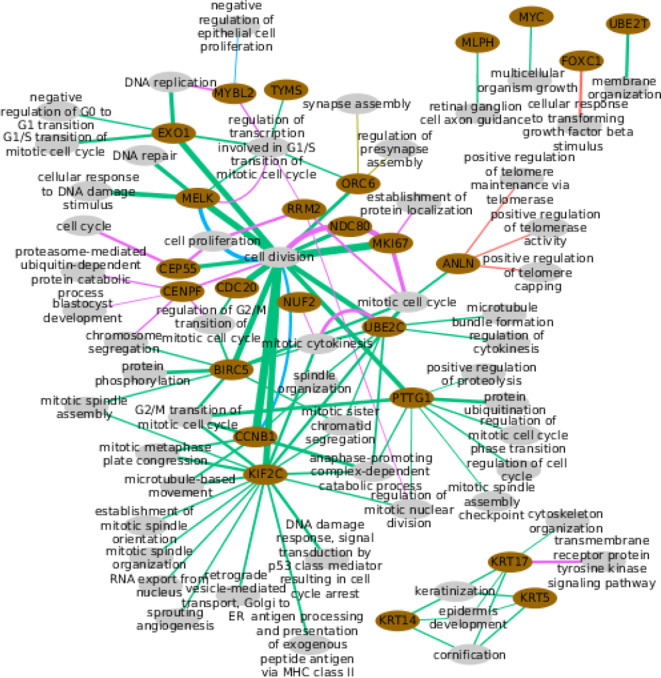
Functional enrichment of the predictors selected per PAM50 gene. Pink ellipses represent PAM50 genes while the gray ellipses represent biological processes. Colored lines link genes with the processes they are significantly enriched to the corresponding subtype. Wider lines indicate a higher number of PAM50 gene predictors in the process.

The selection of predictors we have presented is based on a statistical association with the pattern of expression of a PAM50 gene. The covariation sustaining such an association may respond to how a specific group of predictors is able to attain some biological function. To test this, functional enrichment was done with the set of selected predictors per gene per subtype, versus Gene Ontology Biological Processes categories (GO-BP) (Figure 7).

Only two PAM50 genes are enriched for some process in the Basal subtype, *FOXC1* (basal cluster) and *ANLN*(proliferative cluster). Neither the *ANLN* enrichment for telomere protection nor the *FOXC1* linkage to transforming growth factor response are within these genes immediate annotated processes. Though *FOXC1* is actually related with *TGF*β since both are able to regulate EMT (44).

In the case of Her2+, just *ORC6* (proliferative cluster) is enriched for the totally unexpected process of synapse assembly, but, despite the significant *p*-value, we must notice that this is based on only two genes.

LumA is the most enriched subtype. This is not surprising since it has the largest number of selected coding transcripts, which is the starting material for enrichment. The 20 enriched genes are mostly linked to distinct cellular division aspects. The exception are the three keratins, genes with basal expression, which are connected through their normal processes, suggesting selected predictors respond to the normal gene's function. *MYC* and *UBE2T* are linked to rather wide categories (45) while *MLPH* associates with other than its normal processes. The remaining 14 genes are connected through categories consistent with their proliferative expression, which again alludes to a selection that followed the normal function of the genes. This is again consistent with the available evidence.

For LumB subtype, *MELK* and *CCNB1* enrich for cell division as would be normally expected; while *MYBL2* is unintuitively linked to negative regulation of epithelial cell proliferation, which however, has been reported (46). Finally, the normal tissue shows different cell division aspects coherent with the proliferative expression of its enriched genes.

Altogether, few genes have predictors with significant enrichment extended across subtypes. Eight genes enriched in two subtypes, including *CCNB1, MKI67*, and *UBE2C*, that connect with the same processes, the expected ones, for the two subtypes. *MELK* also connects with its normal process for two subtypes but in LumA and LumB subtypes plus normal tissue. *ANLN, CEP55, KRT17, MYBL2*, and *ORC6*, enrich for different processes across subtypes, that is, a fifth of the genes with any kind of enrichment, but five of the nine genes enriched for more than one subtype.

To further test the functional enrichment per subtype, we compared the sets of predictors selected per subtype for each one of the 9 genes that enrich for several subtypes. Genes enriched for cell division across subtypes, *CCNB1, MKI67*, and *MELK* connect to the process via distinct sets of selected predictors. From the beginning, these genes bear different predictors (Fisher's Exact Test H1: less, *p*-value ≤ 1.281e-09), with a small intersection whose removal does not change the significant enrichment for cell division. This reflects the robustness of the process, which is so important that distinct subsets of the 603 genes annotated in the category are enough to call it.

The other two genes enriched for the same process across subtypes, *UBE2C* for mitotic cytokinesis and, *MELK* for regulation of transcription involved in G1/S transition of mitotic cell cycle, lost the functional enrichment when the predictors selected in both LumA and normal tissue (the intersection) were removed. This implies LumA mitotic cytokinesis and regulation of transcription may be involved in G1/S transition of mitotic cell cycle relying on the normal tissue mechanism.

The quantity of shared predictors between the sets selected for *CEP55*, indicates that predictor selection in the LumA subtype is exclusive for normal tissue selection (Fisher's Exact Test H1: less, *p*-value = 1.141e-10). This means that the differential enrichment between LumA and normal tissue is sustained by different predictors, suggesting *CEP55* fulfills divergent roles in these phenotypes. This matches differences observed between cancer and normal tissue (47) but, to our knowledge, not reported for LumA subtype.

The same reasoning supports *KRT17* and *ORC6* divergent roles across subtypes. It is odd that *KRT17* is linked to kinase signaling for normal tissue and not for a breast cancer subtype, when this has been described for another cancer (48) but this may be associated to tumor incidence over adjacent tissue (49). For *ANLN* and *MYBL2*, selection exclusion between subtypes is not significant, meaning that differential enrichment of these genes could settle on the same predictors, suggesting functional diversity.

## 4. Discussion

Sparse penalized models have already proven useful to discover molecular mechanisms, cluster samples, and predict outcomes such as survival (50). Penalization permits the fitting of models otherwise unattainable given the relatively small sample sizes and huge number of variables measured by the omics. Here, the elastic network approach was used for integrated interpretation of different omics measuring DNA methylation and expression of both coding transcripts and miRNAs.

However, a large training set is always preferable, and not all breast cancer subtypes have been extensively sampled, which is reflected in the models. For Luminal A, the most frequent and sampled subtype, the highest number of predictors were selected by the models; while Her2+, with only 45 samples, got the lowest number of selected predictors. To assure comparability across subtypes we trained the models again, but now using the same number of samples, 40 samples, for all the subtypes. Patterns found with this subset persist in the analysis of the whole set of data, supporting comparability (Figures S5–S8). Nevertheless, the absence of predictors found for LumA in the smaller subtype's models due to a lack of representation can not be ruled out. This could specifically affect the functional enrichment of PAM50 neighborhoods of predictors and so, the functional divergence between subtypes is not definitive but should be experimentally tested.

Multi-omic modeling of PAM50 gene expression is no better than the sole use of coding transcripts, supporting gene expression as the best biomarker of molecular subtypes. However, our point in using the sparse model was not to predict PAM50 but to identify the molecular differences associated with PAM50 signatures that may lead to functional differences.

At the global level, a reduced prediction power of DNA methylation and miRNAs containing models was observed for all subtypes vs. normal tissue, indicating that the influence of this omics on PAM50 gene expression is reduced for cancer. Although this may be born out of incomplete knowledge or incipient technology, an alteration of these omics has been effectively reported; specifically, a generalized hypomethylation has been observed for breast and other cancers (51).

Different predictors were expected per cancer subtype, but the exclusivity of predictors from all the omics was surprisingly high. Only 13 coding transcripts and 2 miRNAs were selected for the four subtypes. The lack of CpGs selected across subtypes is consistent with the high strength of association it has with PAM50 gene expression. If the pattern of expression is different between subtypes, the highly associated CpGs should be different.

The ubiquitous selection of *miR-10b* and *miR-21* across subtypes suggests a central role for these miRNAs in breast cancer, which is actually supported by the literature. Proliferation, cell migration, and *in vivo* tumor growth of MCF7 and MDA-MB-231 cell lines implanted in nude mice is inhibited through antagomiR-21 (52) demonstrating the relevance of this miRNA, at least for luminal A and triple negative subtypes. In turn, both sub and overexpression of *miR-10* are oncogenic. *MiR-10b* overexpression enhances cell migration and invasion by targeting *HOXD10*; while subexpression of *miR-10b-3p*, coded in the same *miR-10b* locus, participates in breast cancer onset by upregulating the cell cycle regulators *BUB1, PLK1*, and *CCNA2* (53).

Coherent with the ubiquitous selection of *miR-21* breast cancer subtypes and its replacement by *miR-10a/b* in normal tissue. *MiR-21* is significantly overexpressed for all cancer subtypes while *miR-10b* is underexpressed, as previous reports say (31). *Mir-10a* is significantly underexpressed in Basal and Her2+ subtypes and slightly overexpressed in luminal subtypes, but this is not significant in LumB case. The proposal is that when *miR-10b* coordinates PAM50 genes, normal tissue expression is predicted; when *miR-10b* is sub expressed and *miR-21* is overexpressed, this second miRNA gains *miR-10b* place, coordinating cancer expression of the PAM50 genes. Since *miR-10b* has a known role in metastasis (31), it would be interesting to observe the dynamics of the networks throughout the evolution of the disease.

Additionally, the small coefficients associated with these miRNAs are consistent with indirect associations. Considering all these pieces, the transition from hub *miR-10a/b* in normal tissue to *miR-21* in breast cancer through the luminal subtypes, evokes a switch between two master regulators. Master regulators are genes needed for the specification of a lineage by its capacity to regulate downstream genes either directly or not, whose missexpression can re-specify the fate of cells (54).

Nonetheless, sparse models can not select regulators naively, they need to feed on known regulators (16, 25, 55). Then, the regulatory capacity of selected predictor can not be stated, leaving *miR-10a/b* and *miR-21* just as universal predictors of PAM50 genes.

Another limitation of the study is the absence of an estimator of significance or accuracy intrinsic to the methodology (56). Regression models quality is described in terms of RMSE, without an indication of how well the selected predictors describe PAM50 expression. A ROC curve is not feasible, since models would have to be turned into the classification setting, and even this is unreachable, because true negative regulators can not be ascertained, as non regulators could simply be regulators yet to discover.

Finally, it is important to mention that applying the same shrinkage to inherently different molecular levels, like CpG methylation and transcript expression, could shrink to zero all the coefficients of subtler effect predictors (13). Thus, the next implementation of sparse multiomic models on PAM50 expression should adopt multiple penalizations, which could even ameliorate the bias on subtype representation (57). Distinct values for the mixing parameter should also be probed, as well as data decomposition into latent variables (58).

###  Future Directions

Apart from exploration of alternative frameworks, the immediate follow up should be the experimental assessment of the observations described here. Specifically, silencing and expression of *miR-10a/b* and *miR-21* need to be tested for each breast cancer subtype. Disection of interaction between the miRNAs and the PAM50 genes is required too.

Then, more omics could be included in the models. Copy number variation is the first candidate to be incorporated since it is already available in the databases and has a proven effect on Her2+ subtype, in particular regarding the effect of the *Her2* amplicon since it has been associated to regulation of growth and survival processes. But single nucleotide variation and chromatin accessibility are also available for some samples.

Other phenotypes with discriminant patterns of expression could benefit from sparse modeling. There could be significant predictors linked to the glioblastoma subtypes as was observed for breast cancer. Predictors represent potential regulators of the mechanisms behind subtype heterogeneity and, as such, are interesting markers of cancer. In this sense, predictor selection across stages, not subtypes, could illuminate the driving forces behind disease development. Alternative methods like A–JIVE (59) and sPLS (60) would have also exciting outcomes in this settings.

A relevant mid to long term future direction will be the implementation of experimental assays to test for multi-omic synergistic or cooperative phenomena, aiming at providing some mechanistic clues of the biological functions behind. There is however a strong challenge on this given the combinatorial mixture of effects that may be complex to disentangle. Some promissory (yet preliminary) advances are starting to arise.

## 5. Conclusion

Holistic studies of cancer are needed to dissect its complexity. Initiatives like The Cancer Genome Atlas have delivered the distinct molecular perspectives that need to be interpreted as a whole. The elastic net models subject of this work, approach such an integration in a rather simplistic linear form. Yet, the methodology is powerful enough to prove the intuition that PAM50 gene expression patterns are accompanied by distinctive potentially regulatory elements. Predictors are selected in an almost exclusive manner, heavily dictated by the omic of origin, with CpGs strongly associated to PAM50 expression not selected across subtypes. The way *miR-10a/b* and *miR-21*, the only relevant predictors selected for all subtypes, are connected and differentially expressed, suggest an specific regulatory difference between breast cancer and normal tissue that merits further research.

## Data Availability Statement

The datasets analyzed for this study can be found in the Genome Data Commons site https://bit.ly/2Itoi2e. The code to perform all previous analyses can be found at the following GitHub repository: https://github.com/CSB-IG/PAM50multiomics.

## Author Contributions

SO organized the database, performed the statistical analysis, and wrote the first draft of the manuscript. GA-J contributed to design of the study, generated programming code, and contributed to the writing of the manuscript. EH-L conceived the study, contributed to design of the study, provided funding, discussed findings, and reviewed the writing of the manuscript. All authors contributed to manuscript revision, read, and approved the submitted version.

## Conflict of Interest

The authors declare that the research was conducted in the absence of any commercial or financial relationships that could be construed as a potential conflict of interest.

## Supplementary Material

Figures S1–S4 depict the topology of the networks for the non-basal subtypes that were not shown. Table S1 contains a list of all validated interactions.
