# Supplementary material for: Multi-Omic Regulation of the PAM50 Gene Signature in Breast Cancer Molecular Subtypes
Source: Front Oncol. 2020 May 22;10:845. doi: 10.3389/fonc.2020.00845 (PMC7259379; doi:10.3389/fonc.2020.00845)
Supplement: Supplementary file 2 [file Data_Sheet_1.PDF]

Supplementary Material

1 SUPPLEMENTARY TABLES AND FIGURES

Table S1. Previously reported predictors to PAM50 gene

1.1 Figures

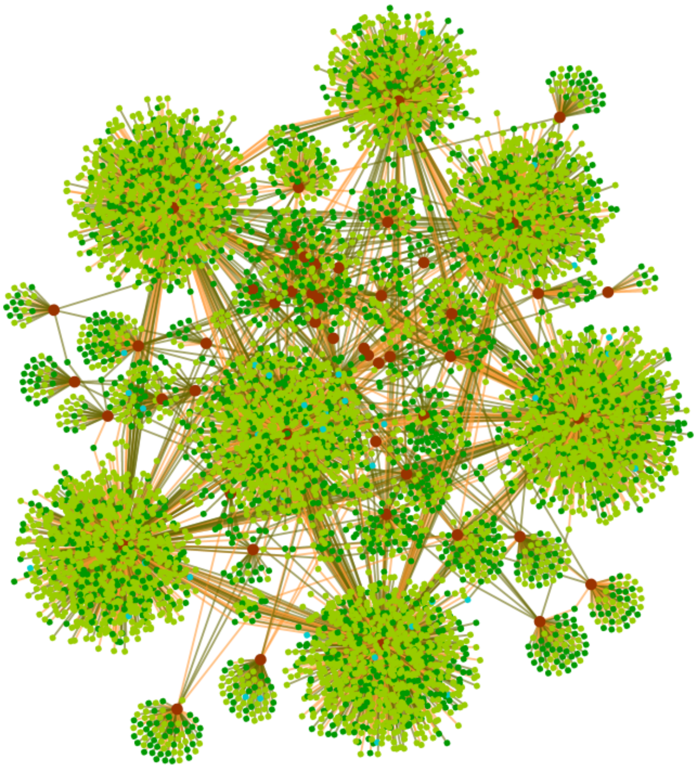

Figure S1. Network of predictors selected for normal tissue.

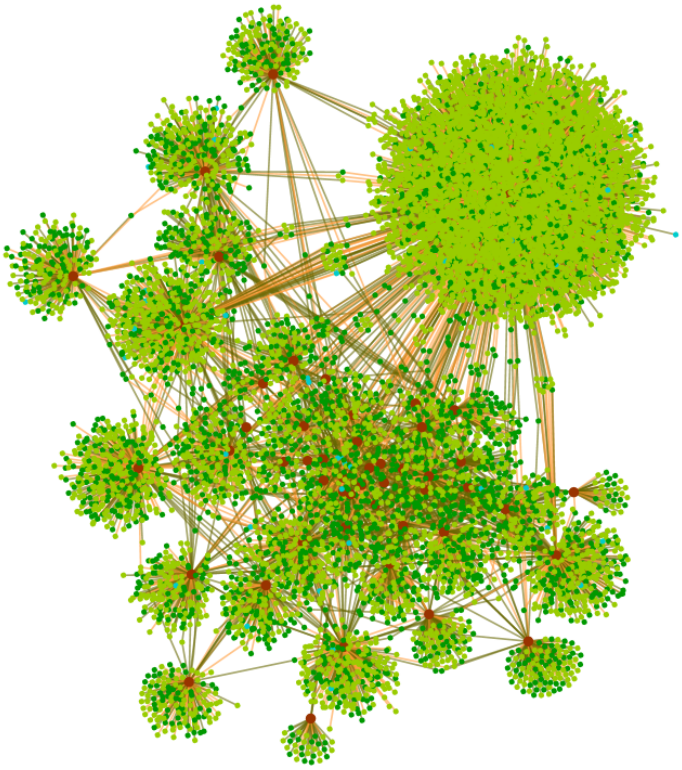

Figure S2. Network of predictors selected for Luminal A subtype.

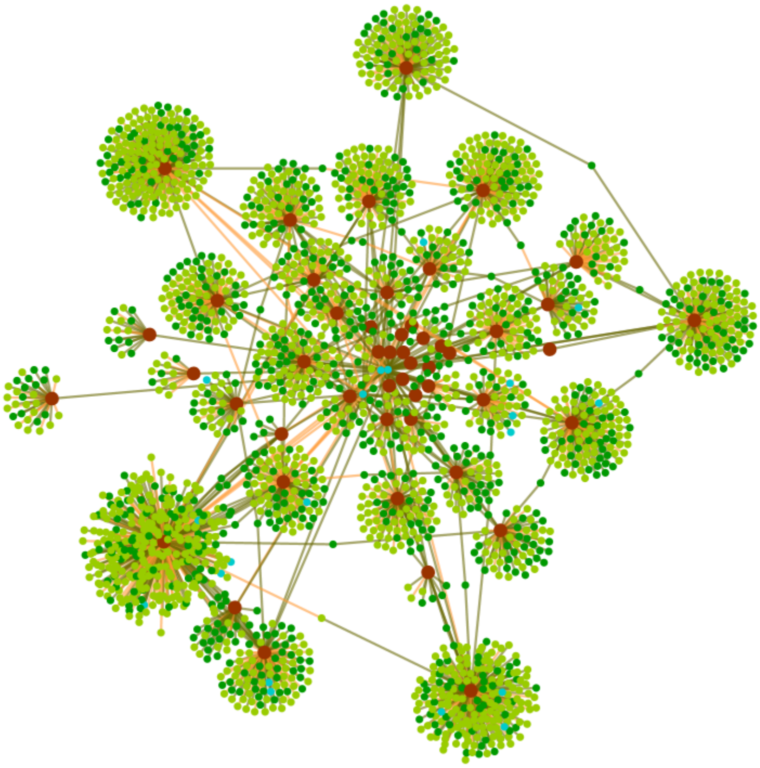

Figure S3. Network of predictors selected for Luminal B subtype.

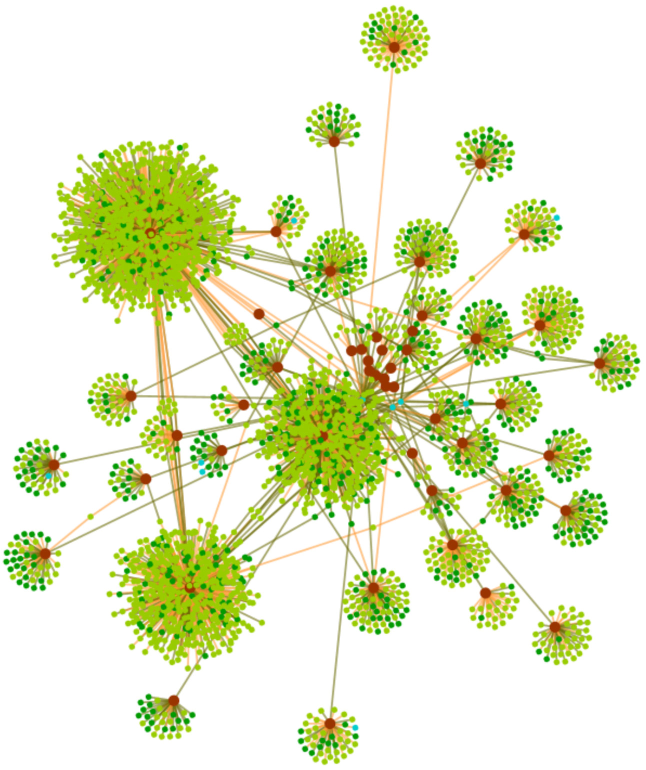

Figure S4. Network of predictors selected for Her2+ subtype.

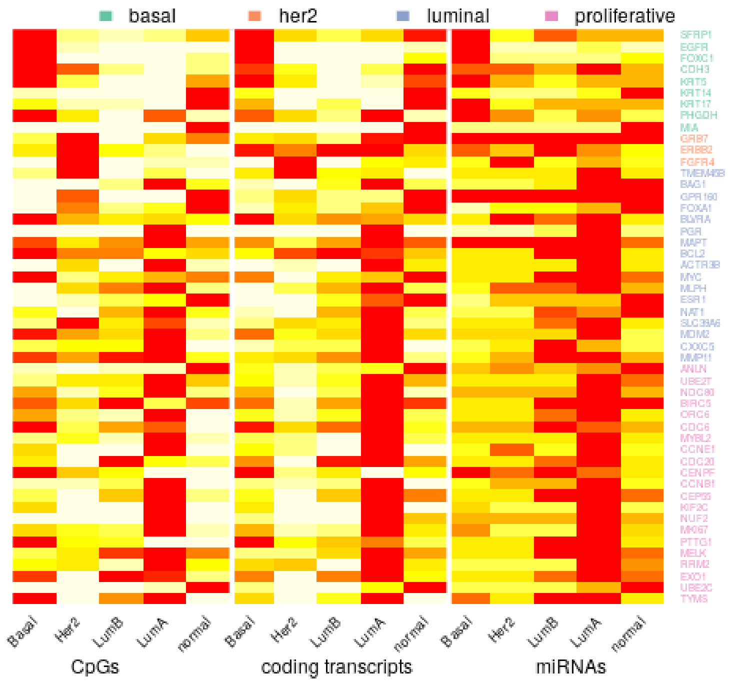

Figure S5. Number of predictors selected per gene and subtype, using the complete dataset.

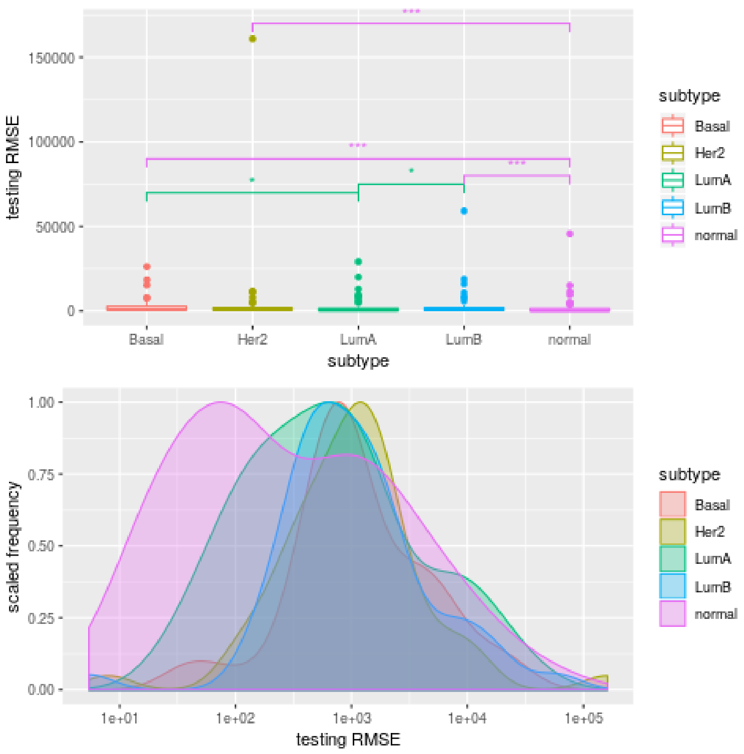

Figure S6. RMSE per subtype using the complete dataset.

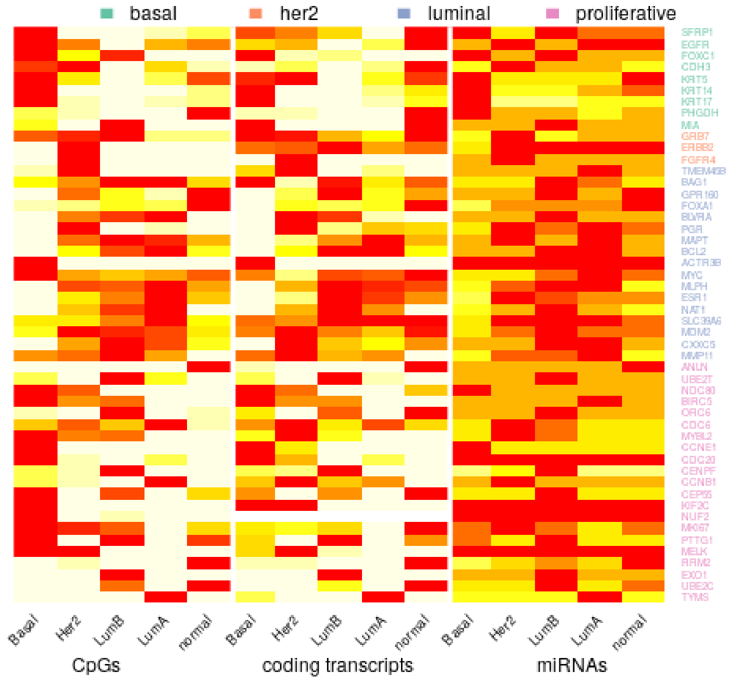

Figure S7. Number of predictors selected per gene and subtype, training only with 40 samples per subtype. PAM50 gene names are indicated at the left and colored by the class of expression.

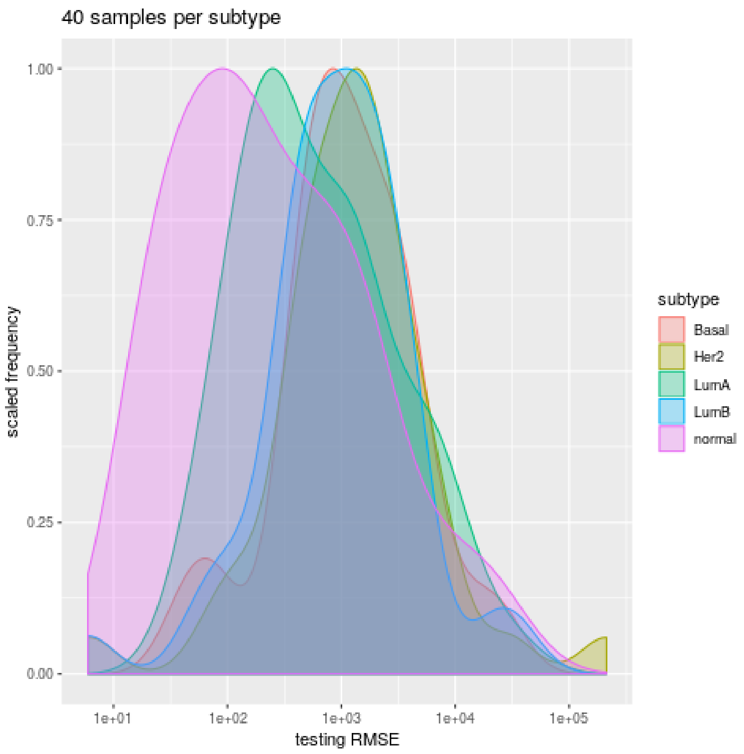

Figure S8. RMSE per subtype training only with 40 samples per subtype.
